# Recent Advances in Substrate-Controlled Asymmetric Induction Derived from Chiral Pool α-Amino Acids for Natural Product Synthesis

**DOI:** 10.3390/molecules21070951

**Published:** 2016-07-21

**Authors:** Seung-Mann Paek, Myeonggyo Jeong, Jeyun Jo, Yu Mi Heo, Young Taek Han, Hwayoung Yun

**Affiliations:** 1 College of Pharmacy, Research Institute of Pharmaceutical Science, Gyeongsang National University, Jinju daero, Jinju 52828, Korea; million@gnu.ac.kr (S.-M.P.); yumiizzi11@naver.com (Y.M.H.); 2 College of Pharmacy, Pusan National University, Busan 46241, Korea; navy7750@gmail.com (M.J.); jju02160@gmail.com (J.J.); 3 College of Pharmacy, Dankook University, Cheonan 31116, Korea; hanyt@dankook.ac.kr

**Keywords:** chiral pool, α-amino acid, natural product, total synthesis, asymmetric induction

## Abstract

Chiral pool α-amino acids have been used as powerful tools for the total synthesis of structurally diverse natural products. Some common naturally occurring α-amino acids are readily available in both enantiomerically pure forms. The applications of the chiral pool in asymmetric synthesis can be categorized prudently as chiral sources, devices, and inducers. This review specifically examines recent advances in substrate-controlled asymmetric reactions induced by the chirality of α-amino acid templates in natural product synthesis research and related areas.

## 1. Introduction

**Figure 1:**
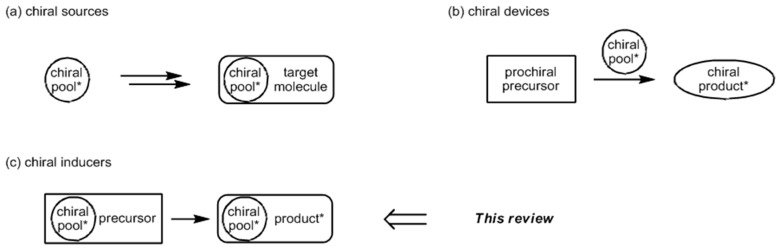
Three categories of chiral pool use in asymmetric synthesis.

The chiral pool approach is highly attractive in the asymmetric total synthesis of bioactive natural products with diverse and complex architectures [1,2]. This strategy is one of the best methods available to synthetic organic chemists for establishing pivotal stereocenters in optically active compounds [3,4,5,6,7]. The chiral pool is a versatile tool, comprising naturally occurring chiral molecules such as carbohydrates, amino acids, terpenes, alkaloids, and hydroxyacids [2,6]. They include enantiomerically enriched species that can be synthetically transformed into the desired target molecules. Chiral pool materials are also inexpensive and commercially available, making them adequate for use in accessing natural products and bioactive compounds [2]. The usage of the chiral pool in asymmetric synthesis can be classified in three general categories, as shown in Figure 1: (a) chiral sources, used as building blocks containing built-in stereocenters for target molecules; (b) chiral devices, employed as useful tools for enantioselective catalysts and auxiliaries; and (c) chiral inducers, applied to the generation of new stereocenters in a substrate-controlled manner [1,2,3,4,5,6,7]. The chiral inducer strategy is a highly efficient method to exploit advantages of both the chiral source and device approach at the same time.

**Figure 2:**
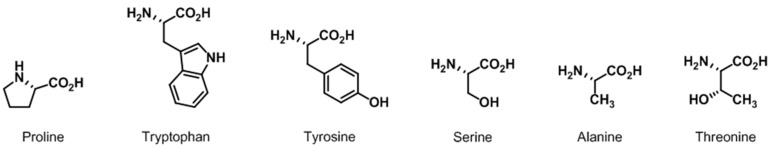
Representative α-amino acids.

The specific aim of this review is to present useful applications of enantiomerically enriched α-amino acids as substrate-controlled asymmetric inducers in natural product synthesis from 2011 to May 2016. Chirally pure α-amino acids are very useful materials due to diversity of functional group and ease of commercial use [7]. The α-amino acids described in this review are illustrated in Figure 2. The use of amino acids as chiral sources and devices for asymmetric synthesis is not covered. Also, synthesis of acyclic or cyclic peptide natural products is not included.

## 2. Chiral Pool: Proline

**Scheme 1:**
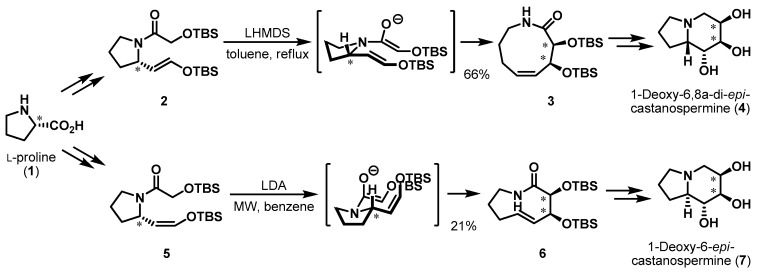
Total syntheses of castanospermines **4** and **7**.

Recently, a wide range of natural and non-natural product syntheses using proline as the chiral pool material in a substrate-controlled manner have been reported. Suh et al. synthesized polyhydroxylated indolizidine alkaloids, 1-deoxy-6,8a-di-*epi*-castanospermine (**4**) and 1-deoxy-6-*epi*-castanospermine (**7**), that can act as selective α-glycosidase inhibitors [8,9]. l-Proline was utilized as a platform to construct the indolizidine skeleton, as shown in Scheme 1. (*E*)-Silyl enol ether **2**, obtained from l-proline via a known protocol [10,11], underwent an aza-Claisen rearrangement to produce the corresponding 9-membered lactam **3** in 66% yield. This transformation was impressive not only because it created a new stereogenic center through a 6-membered transition state, but also because it afforded a *cis*-azoninone framework simultaneously. The final product **4** was afforded after subsequent transformations. Similarly, (*Z*)-silyl enol ether **5** was converted into *trans*-azoninone **6** under microwave-assisted conditions. It is noteworthy that the *syn*-diol moiety of the azoninone skeleton was created via chiral communication of the l-proline stereocenter during aza-Claisen rearrangement-induced ring expansion. The transition states in both these conversions made it possible for the sole chiral center of amino acid **1** to induce additional chirality in *cis* or *trans* azoninones **3** and **6**.

**Scheme 2:**
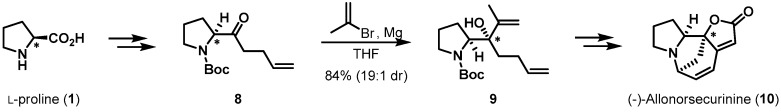
Total synthesis of (−)-allonorsecurinine (**10**).

Another substrate-controlled chiral induction application of l-proline is summarized in Scheme 2. Srihari et al. accomplished the stereoselective total synthesis of alkaloid (−)-allonorsecurinine (**10**) [12]. To create the stereocenter in the lactone moiety of **10**, precursor **8** was readily prepared from l-proline in three steps. The Grignard reaction of isopropenyl magnesium bromide with α-amidoketone **8** afforded tertiary alcohol **9** in high yield and with excellent facial selectivity, with the pivotal tertiary alcohol moiety in **9** constructed via *Si*-face addition to the carbonyl group. With key intermediate **9** in hand, subsequent classical reactions, such as Aldol and Horner–Wittig reactions, provided final product **10**, a Euphorbiaceae alkaloid.

**Scheme 3:**
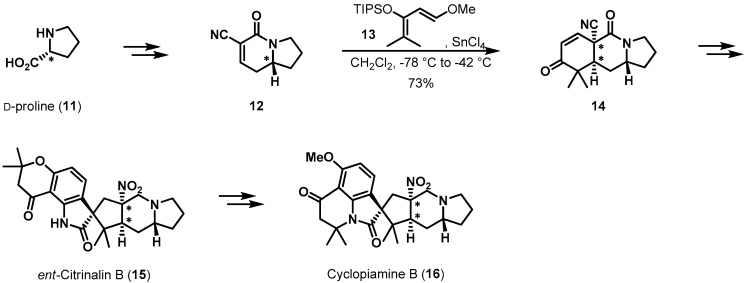
Total syntheses of *ent*-citrinalin B (**15**) and cyclopiamine B (**16**).

Cycloaddition reactions have also been adapted for the proline-derived total synthesis of natural products. Sarpong et al. completed the impressive syntheses of *ent*-citrinalin B (**15**) and cyclopiamine B (**16**) as shown in Scheme 3 [13,14,15]. The authors utilized the chirality of d-proline for the stereoselective construction of a *cis*-fused ring system within final products **15** and **16**. Unsaturated cyanoamide **12** was prepared from d-proline in 55% yield over seven steps for use as a dienophile in the key face-selective Diels-Alder reaction. When diene **13** underwent cycloaddition with **12** in the presence of Lewis acid [16], desired product **14** was obtained in 73% yield after a basic work-up. As dienophile **12** provided a convex face environment in the bicyclic ring system, diene **13** approached the β-face of the unsaturated lactam ring selectively, establishing the two adjacent stereocenters in tricyclic ketone **14** simultaneously. Subsequent steps transformed **14** into *ent*-citrinalin B (**15**) and cyclopiamine B (**16**).

**Scheme 4:**
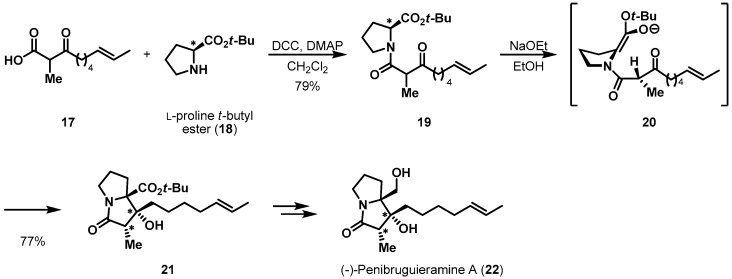
Total synthesis of penibruguieramine A (**22**).

Memory of chirality is a very special case. Recently, Kim et al. reported the first total synthesis of (−)-penibruguieramine A (**22**), employing a biomimetic approach (Scheme 4) [17,18]. Acid **17** was coupled with l-proline *t*-butyl ester (**18**) in the presence of DCC, providing amide **19**, an intramolecular aldol reaction precursor, in 79% yield. Exposure of **19** to sodium ethoxide enabled the pyrrolizidine backbone and two additional stereogenic centers of **21** to be established through memory of chirality and concomitant dynamic kinetic resolution. When amide **19** was treated with a base, its central chirality should have been deleted by deprotonation. However, enolate **20** contained a chiral axis, resulting in memory of chirality and hampering racemization. Impressively, the dynamic kinetic resolution of racemic methine occurred, creating an α-chiral center in the amide moiety. From this transformation, bicyclic amide **21** was obtained in 77% yield, with 10% of the corresponding elimination product. Following an uneventful reduction procedure, (−)-penibruguieramine A was produced from amide **21** in 86% yield (two steps).

## 3. Chiral Pool: Tryptophan

**Scheme 5:**
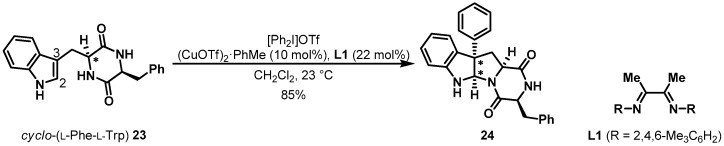
Cu-catalyzed arylation of cyclo-(Trp-Phe) **23**.

Tryptophan, an aromatic amino acid, has been used as a precursor in the total synthesis of natural products with indole-derived heterocyclic framework. The indole moiety of tryptophan serves as a good template for a copper-catalyzed asymmetric arylation, as depicted in Scheme 5 [19]. Reisman et al. investigated and optimized these reaction conditions. After an extensive survey of bidentate ligands and electrophiles under (CuOTf)_2_ PhMe catalyst, cyclic dipeptide **23** in the presence of **L1** and [Ph_2_I]OTf afforded pyrroloindololine **24** in high yield and excellent diastereoselectivity, minimizing the undesired C-2 arylated product. Two newly created stereogenic centers were induced by the chirality of tryptophan in a substrate-controlled manner.

**Scheme 6:**
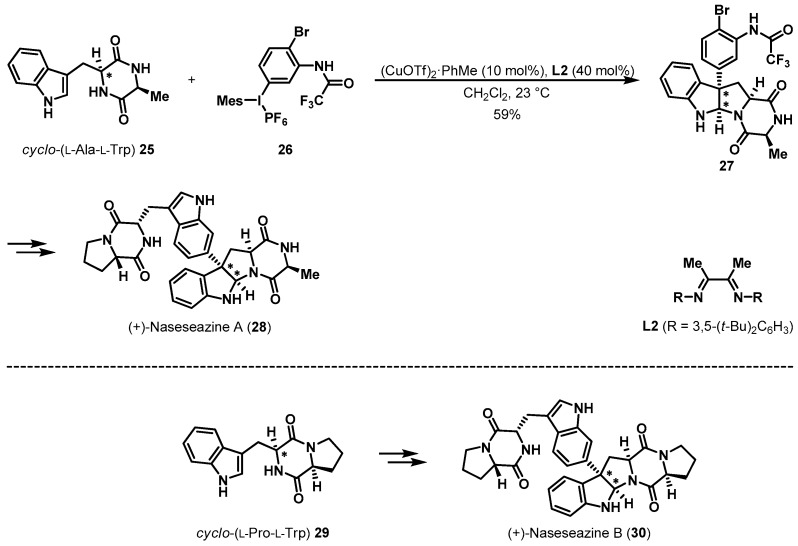
Total synthesis of (+)-naseseazines A and B.

This conversion strategy was applied directly to the total synthesis of (+)-naseseazine A (**28**) and (+)-naseseazine B (**30**) (Scheme 6) [20]. To construct the tetracyclic framework of **28**, cyclic alanine-tryptophan dimer **25** was selected as a chiral precursor. The pivotal arylation of diketopiperazine **25** with advanced electrophile **26** in the presence of (CuOTf)_2_ PhMe and **L2** provided desired pyrroloindoline **27** in moderate yield. Final compound **28** was conveniently constructed from tetracyclic intermediate **27** using a Larock indolization strategy [21,22]. Additionally, another natural product, (+)-naseseazine B, was obtained stereoselectively from cyclic proline-tryptophan precursor **29**, employing a similar synthetic sequence.

**Scheme 7:**
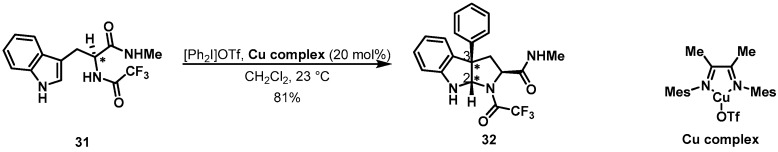
Diastereoselective cyclization for pyrroloindoline skeleton.

Interestingly, applying this methodology to simple carboxamide **31** afforded pyrroloindololine compound **32**, possessing the opposite stereochemistry in the C-2 and C-3 positions. This discrepancy shows that amino acids provide a tremendous opportunity for the diastereoselective synthesis of the pyrroloindololine framework as shown in Scheme 7.

**Scheme 8:**
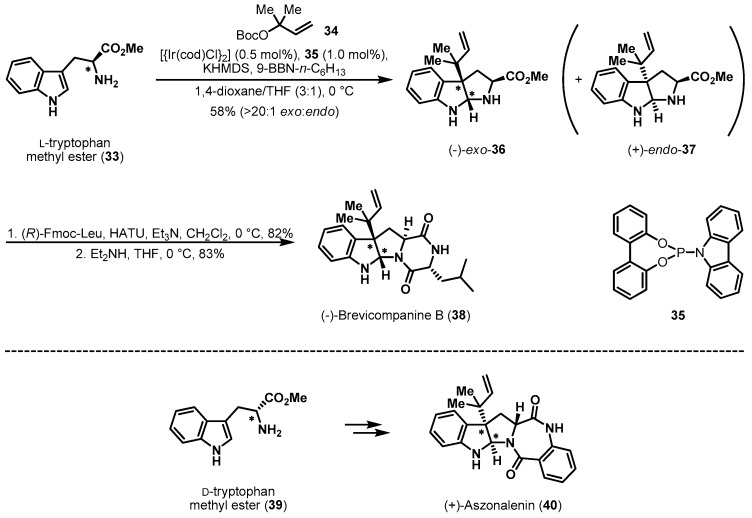
Total syntheses of (−)-brevicompanine B (**38**) and (+)-aszonalenin (**40**).

Tryptophan was also utilized as a chiral pool reagent in the total synthesis of prenylated indole alkaloids (−)-brevicompanine B (**38**) and (+)-aszonalenin (**40**) (Scheme 8) [23]. Carreira et al. reported a highly diastereoselective and regioselective iridium-catalyzed reverse prenylation reaction. The reaction of readily available l-tryptophan methyl ester **33** with tertiary carbonate **34** in the presence of [{Ir(cod)Cl}_2_] and phosphoramidite ligand **35** [24] furnished hexahydropyrrolo[2,3-*b*]indole (−)-exo-**36** in 58% yield. Initially, the *exo*/*endo* ratio of the prenylation was quite low (1.3:1). However, it was improved to >20:1 by extensive optimization of base, ligand, and reaction temperature. Importantly, the installation of two vicinal stereogenic centers was controlled by the chirality of tryptophan. After this successful result, (−)-brevicompanine B (**38**) [25], a plant growth regulator, was finally obtained from iterative amidations in good yield. The total synthesis of another alkaloid, (+)-aszonalenin (**40**) [26], a substance P inhibitor for the human neurokinin-1 receptor, was efficiently completed from d-tryptophan methyl ester **39** via a similar synthetic procedure.

**Scheme 9:**
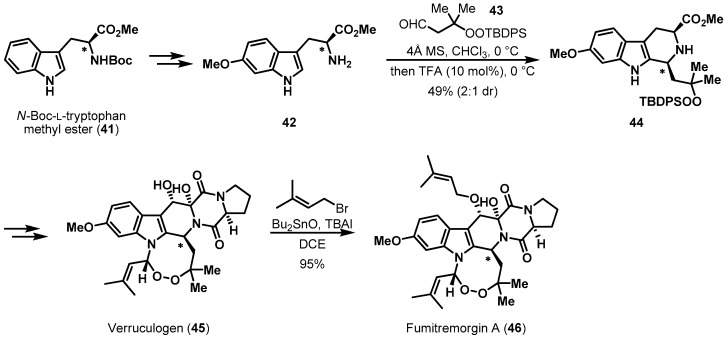
Total syntheses of verruculogen (**45**) and fumitremorgin A (**46**).

A more recent example of tryptophan-templated chiral pool synthesis is illustrated in Scheme 9. Baran et al. accomplished the total syntheses of verruculogen (**45**) and fumitremorgin A (**46**), which both contain a unique eight-membered endoperoxide [27,28,29]. Diastereoselective Pictet-Spengler cyclization of **42**, prepared from *N*-Boc-l-tryptophan methyl ester (**41**), with TBDPS-protected peroxy-aldehyde **43** gave tricycle **44**. Although the facial selectivity was relatively low (2:1), major diastereomer **44** was effectively exploited to finish the total syntheses. The chirality of tryptophan from the chiral pool was critical for creating the new stereocenter in the indole system. The pivotal methoxy group in precursor **42** was introduced by Ir-catalyzed borylation and Chan-Lam coupling [30].

## 4. Chiral Pool: Tyrosine

**Scheme 10:**
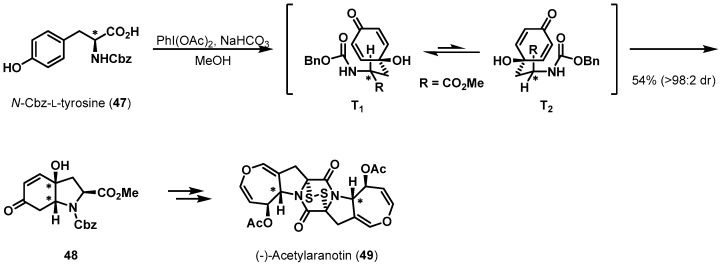
Total synthesis of (−)-acetylaranotin (**49**).

Various natural product syntheses have started from chiral pool reagent tyrosine, which can be transformed into enantiomerically pure intermediates. Tokuyama et al. reported the total synthesis of dimeric alkaloid (−)-acetylaranotin **49** in 2012 (Scheme 10) [31,32]. Alkaloid **49** features a dihydrooxepine backbone synthesized from α,β-unsaturated ketone **48** by olefin isomerization, Wharton rearrangement, Baeyer-Villiger oxidation, and further steps. The total synthesis commenced with the preparation of enone **48** via oxidative dearomatization of *N*-Cbz-l-tyrosine (**47**) and subsequent conjugate addition of the transition state amino moiety. This remarkable reaction was previously developed and described by Wipf et al. [33]. After oxidative dearomatization of the phenol moiety in **47**, two transition states, **T_1_** and **T_2_**, for concomitant conjugate addition are possible. **T_1_** was more stable, due to having less A^1,3^-strain (H and carbonyl oxygen), resulting in **48** being obtained exclusively as the major diastereomer. This high facial selectivity further demonstrates the superiority of amino acids as chiral inducers.

**Scheme 11:**
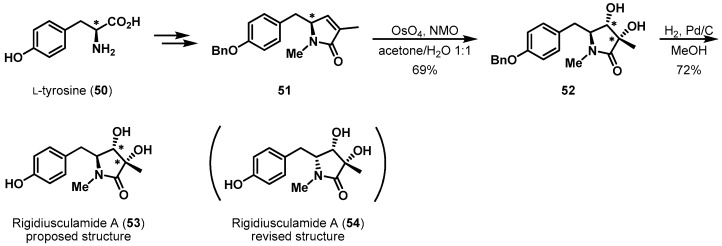
Total synthesis of rigidiusculamide A (**53**).

Alkene asymmetric dihydroxylation is another example of tyrosine utilized as a chiral template. The stereoselective synthesis of pyrrolidinone alkaloid rigidiusculamide A was completed by Krishna et al. (Scheme 11) [34,35]. To incorporate the *syn*-diol moiety in **53**, they dihydroxylated **51** using the Upjohn method (OsO_4_/NMO) [36]. This transformation afforded desired diol **52** as a single diastereomer in 69% yield. The chirality of γ-lactam **52** was thought to be responsible for the enantiomerically pure tyrosine-induced α-facial selectivity. Finally, the *O*-benzyl group in **52** was deprotected to afford the originally proposed structure of rigidiusculamide A. Unfortunately, the experimental data was not identical to that of the authentic natural product [35].

## 5. Chiral Pool: Serine

**Scheme 12:**
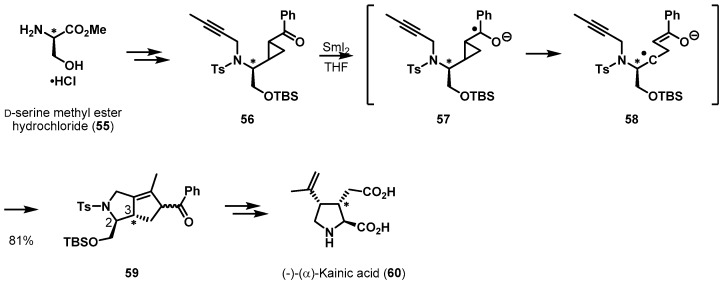
Total synthesis of (−)-α-kainic acid (**60**).

Serine, containing a hydroxymethyl group, has also been used as the powerful chiral pool reagent in the synthesis of complex target molecules. A particularly impressive example, the enantioselective synthesis of (−)-α-kainic acid (**60**), in which Zhou and Li et al. present a unique SmI_2_-catalyzed [3 + 2] intramolecular cycloaddition reaction with excellent diastereoselectivity, is summarized in Scheme 12 [37,38]. The key precursor, cyclopropane **56**, was synthesized from d-serine methyl ester HCl (**55**) using conventional protocols. When cyclopropane **56** was treated with samarium diiodide, ketyl radical **57** was initially formed. Rapid cleavage of the cyclopropyl ring and subsequent cycloaddition was observed, which afforded desired bicyclic ketone **59** in good yield. It was hypothesized that ketyl radical **57** spontaneously transformed into enolate radical **58**. This newly created chiral center favored 2,3-*trans* stereoselectivity over 2,3-*cis* via facial control from the sole chiral center from the chiral amino acid. With key intermediate **59** in hand, the asymmetric synthesis of kainoid **60** was accomplished via a high-yielding sequence.

**Scheme 13:**
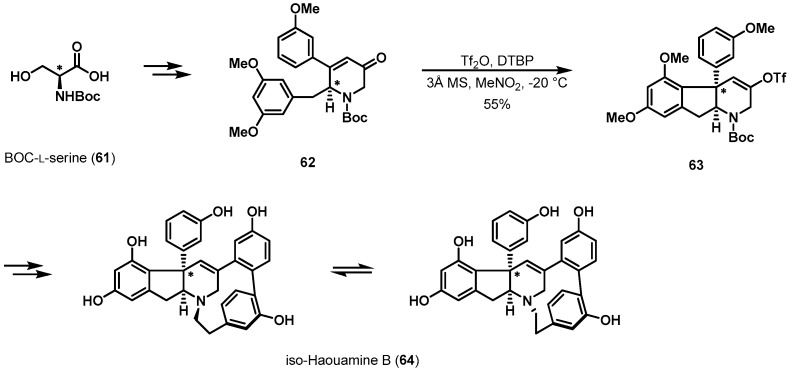
Total synthesis of iso-haouamine B (**64**).

The synthesis of iso-haouamine B is another example (Scheme 13) [39,40]. Structurally, this alkaloid, **64**, consists of an indeno-tetrahydropyridine core fused to a highly distinctive 11-membered *p*-cyclophane ring. Trauner et al. investigated a substrate-controlled oxidative phenol coupling to establish the indeno-tetrahydropyridine ring. Coupling precursor enone **62** was readily prepared from *N*-Boc protected l-serine (**61**). With desired intermediate **62** prepared, carbonyl activation by triflic anhydride and concomitant 1,4-addition of the electron-rich aromatic ring produced enol ether **63** in moderate yield. During the crucial addition process, the *syn*-substituted cyclopentane skeleton was constructed without racemization. The new quaternary stereogenic center in **63** was ultimately derived from the chiral pool stereocenter in **62**.

**Scheme 14:**
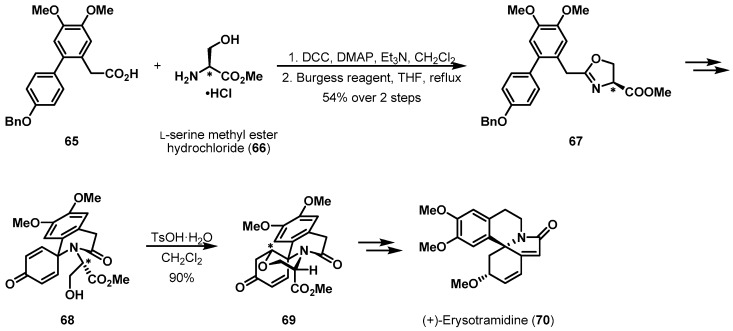
Total synthesis of (+)-erysotramidine (**70**).

More recently, Ciufolini et al. described the total synthesis of (+)-erysotramidine (**70**) using an l-serine derivative (Scheme 14) [41,42]. Advanced oxazoline **67**, prepared from l-serine methyl ester (**66**) via DCC coupling and Burgess reagent-induced cyclization [43], was converted into enone **68** as a precursor for stereoselective Michael cyclization. Exposure of electronically deficient enone **68** in CH_2_Cl_2_ to TsOH resulted in the desired tetracyclic core of **69** as a single diastereomer in excellent yield. Although the serine hydroxyl group could approach the two reactive Michael acceptors, the formation of desired product **69** was favored. The high diastereoselectivity was assumed to result from the minimization of unwanted nonbonding interactions between the methyl ester group and acylamido group [44,45]. The pseudoaxial conformation of the methyl ester group resulted from the chirality of serine. After this chiral communication, (+)-erysotramidine (**70**) was synthesized from key synthetic intermediate **69** using further manipulations.

## 6. Chiral Pool: Alanine

**Scheme 15:**
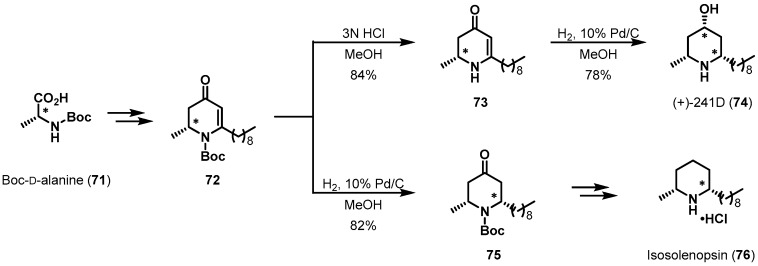
Total syntheses of (+)-241D (**74**) and isosolenopsin (**76**).

The stereocenter in alanine, a simple chiral pool reagent, has also provided good opportunities for chiral induction. Gouault et al. accomplished the asymmetric total synthesis of dendrobate alkaloid (+)-241D (**74**) and isosolenopsin (**76**) (Scheme 15) [46,47,48]. Structurally, these alkaloids consist of *cis*-2,6-dialkylpiperidine. Vinylogous lactams **72** and **73**, which were used as chiral precursors, were readily prepared from *N*-Boc protected d-alanine **71** [49]. The catalytic hydrogenation of Boc-deprotected amine **73** finally gave the target molecule, (+)-241D (**74**), via stereoselective reduction of both the alkene and ketone. In addition, key intermediate **75**, which was transformed into enantiopure alkaloid isosolenopsin (**76**), was obtained by hydrogenation under similar conditions. The newly generated stereogenic centers in **74** and **75** were affected by the chirality of d-alanine. The total synthesis of isosolenopsin (**76**) was completed using deoxygenation and Boc-deprotection steps.

## 7. Chiral Pool: Threonine

**Scheme 16:**
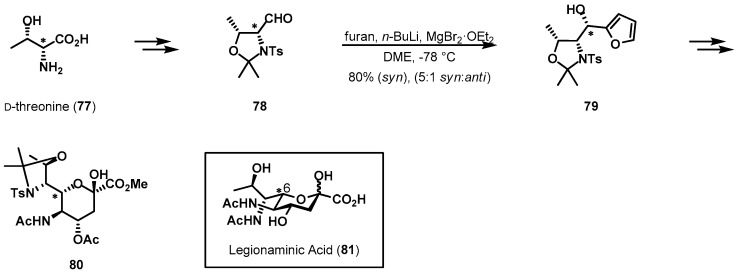
De novo synthesis of orthogonally protected legionaminic acid **80**.

α-Amino acid threonine is a special chiral pool reagent, containing an extra stereocenter. Recently, Seeberger et al. published the total synthesis of protected legionaminic acid **80** from d-threonine as a starting material (Scheme 16) [50,51]. Conventional protection of chiral pool reagent **77**, followed by DIBAL-H reduction, provided chiral aldehyde **78** in high yield [52]. With threoninal **78** in hand, treatment with 2-lithiofuran resulted in desired alcohol **79**. Although the organometallic addition reaction could produce a diastereomeric mixture, the desired *syn*-configured alcohol, **79**, was obtained with a 5:1 ratio and in 80% isolated yield. This stereoselectivity was caused by the chirality of threonine amino acid via Cram-chelation control of the nucleophilic addition [53]. The newly generated stereogenic center served as a key stereocenter in C-6 within legionamic acid (**81**).

## 8. Conclusions

Naturally occurring chiral pool α-amino acids provide synthetic chemists with a powerful tool for the incorporation of pivotal stereocenters in optically active natural products. Until now, α-amino acids have been exploited for use not only as chiral sources and devices, but also as chiral inducers in strategies for the synthesis of complex target molecules. In this review, many applications of α-amino acids as chiral inducers in a substrate-controlled manner were specifically discussed. To establish challenging stereocenters in natural product architectures, the chirality of α-amino acids was applied to a remarkable variety of reactions, such as rearrangement, cyclization, cycloaddition, nucleophilic addition to carbonyls, and hydrogenation. To conclude, attempts at utilizing α-amino acids as chiral inducers for the creation of new stereogenic centers will continue.

## Abbreviations

The following abbreviations are used in this manuscript:

Ac	Acetyl
Ala	Alanine
BBN	Borabicyclo[3.3.1]nonane
Bn	Benzyl
Boc	*t*-Butoxycarbonyl
Bu	Butyl
Cbz	Benzyloxycarbonyl
cod	1,5-Cyclooctadiene
DCE	1,1-Dichloroethane
DCC	*N,N*'-Dicyclohexylcarbodiimide
DIBAL-H	Diisobutylaluminum hydride
DMAP	*N,N*-4-Dimethylaminopyridine
DME	1,2-Dimethoxyethane
DTBP	2,6-Di-*tert*-butylpyridine
Et	Ethyl
Fmoc	9-Fluorenylmethoxycarbonyl
HATU	*O*-(7-azabenzotriazol-1-yl)-*N*,*N*,*N*’,*N*’-tetramethyluronium hexafluorophosphate
KHMDS	Potassium *bis*(trimethylsilyl)amide
LDA	Lithium diisopropylamide
Leu	Leucine
LHMDS	Lithium *bis*(trimethylsilyl)amide
Me	Methyl
Mes	Mesityl
MS	Molecular sieves
MW	Microwave
NMO	*N*-Methylmorpholine *N*-oxide
Phe	Phenylalanine
TBAI	Tetra-*n*-butylammonium iodide
TBDPS	*t*-Butyldiphenylsilyl
TBS	*t*-Butyldimethylsilyl
Tf	Trifluoromethanesulfonyl
TFA	Trifluoroacetic acid
THF	Tetrahydrofuran
TIPS	Triisopropylsilyl
Trp	Tryptophan
Ts	*p*-toluenesulfonyl
